# Inter-observer agreement and image quality of model-based algorithm applied to the Coronary Artery Disease-Reporting and Data System score

**DOI:** 10.1186/s13244-022-01286-5

**Published:** 2022-11-18

**Authors:** Davide Ippolito, Cammillo Talei Franzesi, Cecilia Cangiotti, Luca Riva, Andrea De Vito, Davide Gandola, Cesare Maino, Paolo Marra, Giuseppe Muscogiuri, Sandro Sironi

**Affiliations:** 1 Department of Diagnostic Radiology, San Gerardo Hospital, Via Pergolesi 33, 20900 Monza, MB Italy; 2 School of Medicine, University of Milano-Bicocca, Via Cadore 48, 20900 Monza, MB Italy; 3 Department of Diagnostic Radiology, H Papa Giovanni XXIII, Piazza OMS 1, 24127 Bergamo, BG Italy; 4 IRCCS, Istituto Auxologico Italiano, Milan, Italy

**Keywords:** Coronary artery disease, Coronary Artery Disease-Reporting and Data System, Radiation exposure, Cardiac computed tomography angiography, Model-based iterative reconstruction algorithm

## Abstract

**Purpose:**

To evaluate the inter-observer agreement of the CAD-RADS reporting system and compare image quality between model-based iterative reconstruction algorithm (MBIR) and standard iterative reconstruction algorithm (IR) of low-dose cardiac computed tomography angiography (CCTA).

**Methods:**

One-hundred-sixty patients undergone a 256-slice MDCT scanner using low-dose CCTA combined with prospective ECG-gated techniques were enrolled. CCTA protocols were reconstructed with both MBIR and IR. Each study was evaluated by two readers using the CAD-RADS lexicon. Vessels enhancement, image noise, signal-to-noise (SNR), and contrast-to-noise (CNR) were computed in the axial native images, and inter-observer agreement was assessed. Radiation dose exposure as dose–length product (DLP) and effective dose were finally reported.

**Results:**

The reliability analysis between the two readers was almost perfect for all CAD-RADS standard categories. Moreover, a significantly higher value of subjective qualitative analysis, SNR, and CNR in MBIR images compared to IR were found, due to a lower noise level (all *p* < 0.05). The mean DLP measured was 63.9 mGy*cm, and the mean effective dose was 0.9 mSv.

**Conclusion:**

Inter-observer agreement of CAD-RADS was excellent confirming the importance, the feasibility, and the reproducibility of the CAD-RADS scoring system for CCTA. Moreover, lower noise and higher image quality with MBIR compared to IR were found.

**Implications for practice:**

MBIR, by reducing noise and improving image quality, can help a better assessment of CAD-RADS, in comparison with standard IR algorithm.

## Keypoints

- CAD-RADS can improve communication between radiologists and cardiologists and suggest clinical management.
- The MBIR algorithm allows to perform low-dose examinations (80 kV), improving the image quality
- The MBIR offers higher qualitative and quantitative images in the evaluation of coronary arteries, compared with IR algorithm

## Introduction

In the last years, computed tomography (CT) technology has shown essential changes and improvements, and cardiac CT angiography (CCTA) has rapidly evolved as the most non-invasive test in the detection of coronary artery disease (CAD) in low- to intermediate-risk patients [1].

The progressive expansion from 64 to 320 slices allowed an increment of spatial and temporal resolution, leading to a more precise evaluation of atherosclerosis plaque composition (calcified, non-calcified, and mixed plaque). Moreover, CCTA can detect subclinical CAD, positive vessel remodeling, and spotty calcifications [2], in addition to the evaluation of extracardiac findings.

The 2016 updated National Institute for Health and Care Excellence (NICE) guidelines removed the pre-test probability model and evaluated the diagnostic accuracy of the non-invasive test against invasive coronary angiography (ICA) for significant stenosis detection, suggesting CCTA as the first-line test in CAD positive patients [3, 4]. A recent meta-analysis of diagnostic accuracy of CCTA demonstrated an overall sensitivity of 99% and specificity of 87%, compared to ICA as the reference standard, with an added high negative predictive value (up to 100%) [5, 6].

SCOT-HEART and PROMISE studies have also shown the usefulness of CCTA in addition to or as an alternative to functional testing, respectively [7, 8]. Essentially the new European Society of Cardiology (ESC) guidelines give greater prominence to CCTA to confirm CAD; in particular, in case of obstructive CAD that is not ruled out with clinical assessment, CCTA is equally recommended as an alternative initial approach. However, it has been reported that post-CCTA patient management is often sub-optimal [1, 9].

With the large incidence of CAD and therefore of patients undergoing CCTA, a high level of expertise for image interpretation and standardization in the reports are required [10]. The society of cardiovascular computed tomography (SCCT), the American college of radiology (ACR), and the North American society of cardiovascular imaging (NASCI) drew up the Coronary Artery Disease-Reporting and Data System (CAD-RADS) score, to help improve communication between radiologist and cardiologist and suggesting subsequent clinical management [11, 12].

In these settings, CCTA became a widespread diagnostic tool in CAD patients and, consequently, dose reduction should be mandatory according to the ALARA principle. Nowadays, this is possible due to the introduction in clinical practice of the new model-based iterative reconstruction (MBIR) algorithms that allow to perform low-dose examinations, reduce image noise, and lead to equal or better diagnostic quality compared to standard-dose CT reconstructed with iterative reconstruction (IR) algorithm.

On this basis, this study aims to assess the inter-observer agreement of the CAD-RADS reporting system of low-dose CCTA and compare the subjective and objective image quality between the MBIR and the IR algorithms.

## Material and methods

### Study population

This retrospective study conformed to the ethical guidelines of the 1975 Declaration of Helsinki, and the protocol was approved by the institutional review board with a waiver of written informed consent. This was a retrospective observational analysis based on previously collected routine care data. All radiological and clinical data have been anonymized before being analyzed.

All patients with clinical suspicion of obstructive CAD between January 1st, 2020, and December 31st, 2020, were retrospectively included.

Exclusion criteria were: (1) patients with unstable angina, (2) patients with severe renal failure (eGFR < 30 mL/min/1.73 m^2^) or other contraindications for iodinated contrast material (i.e.previous allergic reaction), (3) heart rate > 85 bpm with contraindications to the use of β-blocker, (4) presence of arrhythmia or atrial fibrillation, (5) unstable clinical condition, (6) inability to perform a breath-hold, (7) patients underwent CCTA with poor or non-diagnostic image quality.

**Fig. 1:**
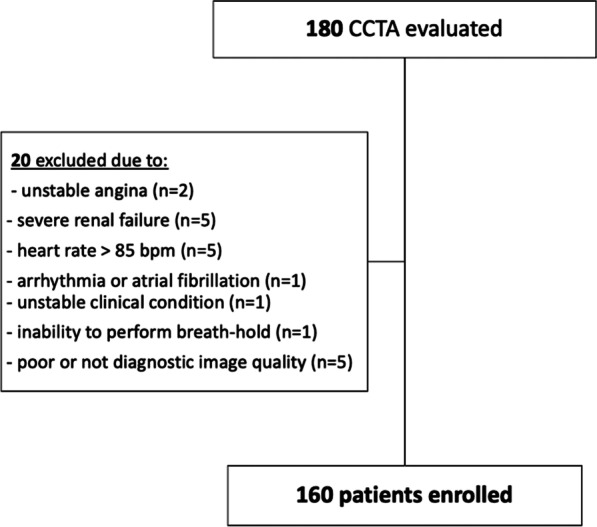
Flowchart of the study

Flowchart in Fig. 1 summarizes the enrollment process.

### CCTA protocol

All patients underwent low-kV CCTA protocol combined with a prospective ECG-gated technique by using a 256-MDCT scanner (iCT Elite, Philips Medical Systems, Best, The Netherlands), a dual-mode scout (on the coronal and sagittal plane) to select the acquisition volume with the lowest scan length according to the patient’s anatomy.

The 80 kV setting increased to 100 kV for patients with BMI over 30. Automated tube-current modulation, with a pre-set value depending on the patient's shape and geometry from the scout image, and a dose right index of 7 was used to reduce the total radiation dose. The other scan parameters were as follows: collimation 128 × 0.625; rotation time 0.27 s; thickness 0.67 mm; increment 0.335 mm; FOV 250 mm; matrix 512 × 512.

CT data were acquired with step and shoot acquisition with a prospective ECG-triggered axial acquisition, selecting the 78% (± 3%) of the cardiac cycle (R-R interval).

In each patient, an 18-gauge intravenous cannula was placed in an antecubital vein of the upper limb, and the contrast medium (CM) was injected using an automatic double-syringe injector (Medrad Stellant, Pittsburgh, PA, USA). A standardized volume of contrast medium of 70 ml (Iobidtritol 350—Xenetix, Guerbet, Aulnay, France) with a flow rate of 4.5 mL/s followed by saline flushing (volume 50 ml, flow rate 4.5 mL/s) was used.

If the baseline heart rate (HR) was > 65 beats per minute (bpm) and patients had no contraindications for β-blockers, metoprolol (5–20 mg) was injected intravenously before the examination.

The start of scanning was obtained for each patient by using a bolus-tracking technique, with a trigger area manually placed at the proximal ascending aorta with a threshold of 120 HU and an 8-s delay.

All raw data were reconstructed with the standard filter "Cardiac Routine" with both algorithms: MBIR (IMR, Level 1, Philips Healthcare, Cleveland, OH, USA) and IR (iDose, Level 4, Philips Healthcare, Cleveland, OH, USA). CCTA protocol is summarized in Table 1.

**Table 1:** Scanning parameters and reconstruction algorithms used

CT scan parameters	MBIR	IR
Tube-voltage (kV)	80	
Tube-current (mAs)	Automated	
Gantry rotation time (s)	0.27	
Detector configuration	128 × 0.625	
FOV (mm)	250	
Thickness; increment (mm)	0.67/0.34	1.0/1.0
CM volume (mL); flow rate (mL/s)	60/4.5	60/4.5

*kV* kilovoltage, *mAs* milliampere-seconds, *FOV* field-of-view, *MBIR* model-based iterative reconstruction, *IR* iterative reconstruction

### Image analysis

Images were processed on a dedicated workstation (IntelliSpace Portal 9.0, Philips) to compute multiplanar reconstructions (MPR), maximum intensity projections (MIP), and volume rendering (VR) images. The CAD-RADS assessment categories and modifiers [10], quantitative and qualitative images analyses were performed by two radiologists with 4 (reader 1) and 7 years of experience (reader 2) in CCTA, CTA, and 3D vascular images interpretation, blinded each other and to clinical data.

#### Qualitative image evaluation

The diagnostic image quality of the ascending aorta and the coronary arteries (RCA, CTk, LAD, and LCx) was evaluated using a 5-point Likert scale for coronary CTA by the two readers, based on the presence of motion artifacts and image noise influencing subjective image quality, as follows: 5 = excellent image quality, 4 = good image quality, 3 = acceptable image quality, 2 = below-average image quality, 1 = poor image quality.

#### Quantitative image evaluation

Each study was evaluated using the CAD-RADS lexicon based on the degree of maximum coronary stenosis among vessel segments larger than 1.5 mm in diameter. All vessels were evaluated with a scoring system from 0 to 5, as follows: 0 = absence of atherosclerosis, 1 = minimal stenosis or plaque with no stenosis (1–24%), 2 = mild stenosis (25–49%), 3 = moderate stenosis (50–69%), 4A = severe stenosis (60–79%) or 4B = left main > 50% or 3 vessel obstructive (> 70%), 5 = total occlusion (100%).

Moreover, CAD-RADS categories were integrated by modifiers as follows: N = non-diagnostic study, S = presence of a stent, G = presence of graft, and V = presence of vulnerable plaque [11].

**Fig. 2:**
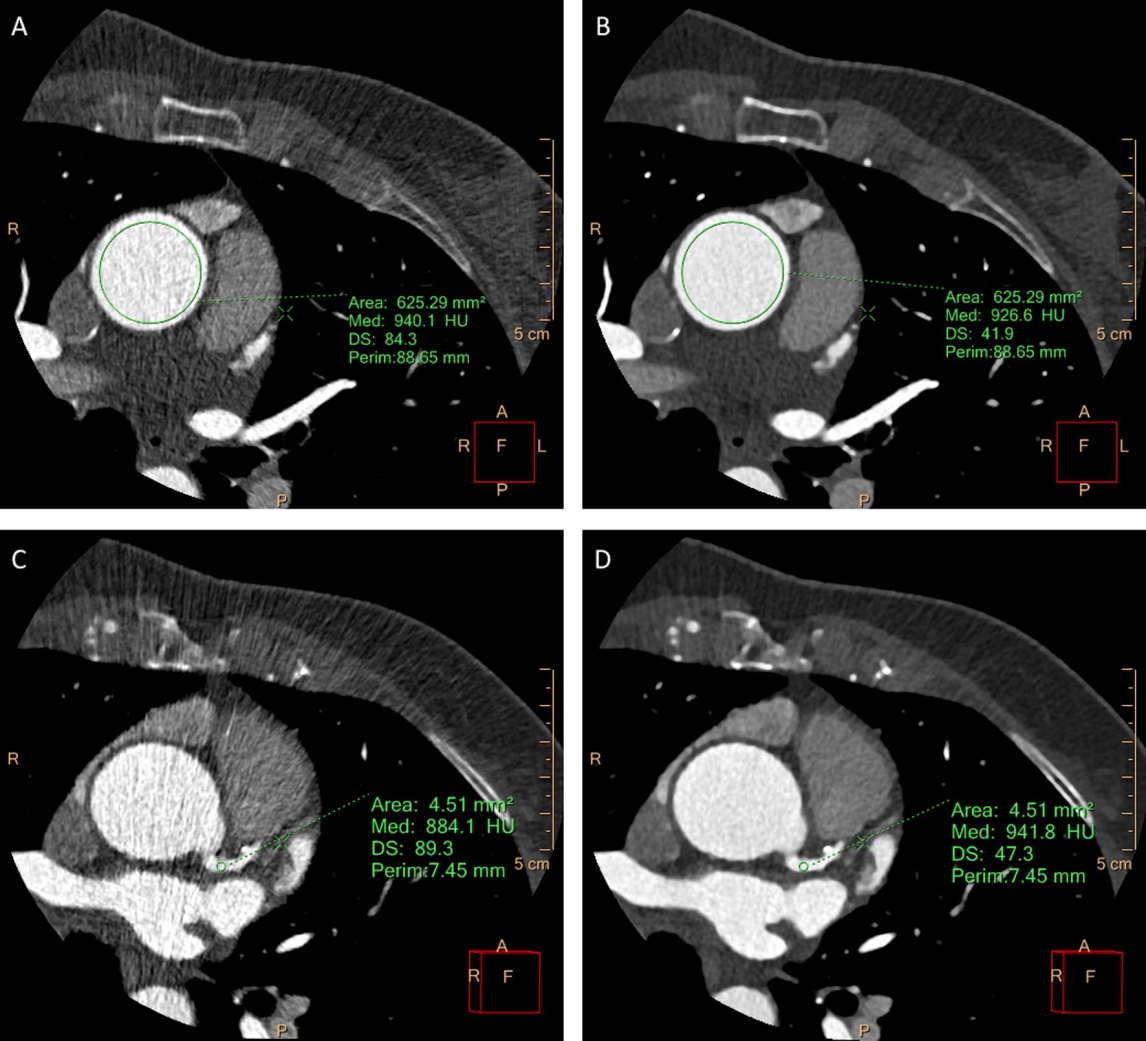
Evaluation of noise and HU of the same patient’s examination dataset reconstructed with the two different iterative algorithms, model-based (MBIR) and hybrid-iterative (IR). CCTA was acquired with an 80-kV protocol, with a low radiation dose exposure (DLP 98.5 mGy × cm; ED 1.44 mSv), with a CADARAD score of 0. **a**–**c** Axial images reconstructed with IR with circular ROI placed in the proximal ascending aorta and common trunk. **b**–**d** Axial images reconstructed with MBIR with circular ROI placed in the same position as figures A and C. Because of the use of the model-based iterative reconstruction algorithm (MBIR), we obtained an image noise reduction of 50% compared to IR reconstruction as reported in the images: IR standard deviation (SD) in aorta 84 and common trunk 89; MBIR SD: aorta 42 and common trunk 47

Vessel contrast enhancement (mean attenuation value, HU) and image noise, defined as the standard deviation of the attenuation values (SD) (Fig. 1), were measured by manually placing a circular region of interest (ROI) at the center of the vascular lumen in the ascending aorta (AO), in the proximal segment of right coronary artery (RCA), common trunk (CTk), left anterior descending (LAD) and left circumflex (LCx) (Fig. 2).

The signal-to-noise ratio (SNR) was computed using the formula SNR $=\frac{{\mathrm{HU}}_{\mathrm{A}}}{{\mathrm{SD}}_{\mathrm{A}}}$, where HU_A_ is the mean attenuation of the coronary artery (at each proximal segment) and SD_A_ is the standard deviation of the HU values. Finally, the contrast resolution was evaluated by calculating the contrast-to-noise ratio (CNR) using the formula CNR $=\frac{{\mathrm{HU}}_{\mathrm{A}}-{\mathrm{HU}}_{\mathrm{B}}}{{\mathrm{SD}}_{\mathrm{B}}}$, where HU_A_ is the attenuation of the proximal tract of coronary arteries and the ascending aorta and HU_B_ and SD_B_ are the attenuation and standard deviation of the adjacent adipose tissue, as previously reported [13, 14].

### Radiation dose

CT dose index (CTDIvol, mGy) and CT dose-length product (DLP, mGy·cm) were registered for all examinations. The effective dose (ED) was computed using the formula ED = k × DLP, where k is the region-specific normalized effective dose (mSv/mGycm) derived from the paper by Deak et al. [14]. A *k* value of 0.0146 mSv/mGy-1·cm-1 [15] was adopted to estimate the effective dose from cardiovascular imaging procedures for adult patients.

### Statistical analysis

Continuous variables were expressed as means and standard deviations and compared by using the Mann–Whitney test or t-Student’s test, when appropriate.

The agreement between the two readers was assessed using the Cohen kappa or Weighted kappa coefficients (0.00–0.20 indicates slight agreement; 0.21–0.40, fair agreement; 0.41–0.60, moderate agreement; 0.61–0.80, substantial agreement; and 0.81–1.00, almost perfect agreement), in case of 2 or more than 2 categorical variables, respectively.

The comparison between the continuous variables measured by the two readers was assessed with Spearman correlation and the Bland–Altman Limits of Agreement (LoA) with the 95%CIs.

A *p*-value < 0.05 was considered significant. The analysis was performed using SPSS software (v 26.0, SPSS Inc, Chicago, Illinois).

## Results

### Study population

A total of 160 patients (M/F = 98/62) with a mean age of 68 ± 9 years (range 33–78) were retrospectively enrolled. Table 2 summarizes the collected clinical data.

**Table 2:** Clinical data of enrolled patients

N = 160	
Sex, male (*n*, %)	98 (61.3)
Age, years (mean ± SD)	68 ± 9
History of CAD (*n*, %)	31 (19.4)
Clinical symptoms (*n*, %)	72 (45.7)
Positive stress echocardiogram test (*n*, %)	20 (12.5)
Previous stent placement (*n*, %)	14 (8.8)
Previous graft placement (*n*, %)	10 (6.3)
Heart rate, bpm (mean ± SD)	55 ± 4
R-R interval, s (mean ± SD)	0.9 ± 0.2

*CAD* coronary artery disease, *SD* standard deviation

All CCTA examinations were completed in a single breath-hold, without any complications and any adverse event after CM injection.

### Image analysis

#### Qualitative analysis

The inter-observer agreement of the subjective image quality evaluation was good (*k* = 0.630). The overall image quality evaluation of the two reconstruction algorithms demonstrated a statistically significant higher score in MBIR images compared to IR, for the two readers [reader 1: MBIR 4 (IQR: 3–4) vs. IR 3 (2–3), *p* < 0.000; reader 2: MBIR 4 (3–4) vs. IR 3 (2–3), *p* < 0.0001].

#### Quantitative analysis

The mean attenuation value of RCA, CTk, and LAD arteries in MBIR images was significantly higher compared to IR (Table 3). Also, the LCx artery and AO demonstrated a higher intra-vessel density value, without a statistically significant difference (*p* = 0.122 and *p* = 0.445, respectively). Moreover, the CNR and SNR were higher in MBIR compared to IR images, with a statistically significant difference (*p* < 0.0001), as shown in Table 3.

**Table 3:** Comparison between MBIR and IR for HU, SD (noise), CNR and SNR. Comparisons were computed using t-Student’s test

Vessel	HU (± SD)			SD (± SD)			CNR (± SD)			SNR (± SD)		
	MBIR	IR	*p*-value	MBIR	IR	*p*-value	MBIR	IR	*p*-value	MBIR	IR	*p*-value
RCA	500 ± 123	474 ± 126	** < 0.0001**	19 ± 6	26 ± 7	** < 0.0001**	28 ± 13	18 ± 8	** < 0.0001**	27 ± 20	13 ± 6	** < 0.0001**
CTk	477 ± 119	466 ± 131	**0.032**	25 ± 8	29 ± 10	** < 0.0001**	26 ± 12	18 ± 8	** < 0.0001**	24 ± 11	12 ± 6	** < 0.0001**
LAD	506 ± 119	489 ± 132	**0.034**	18 ± 8	27 ± 9	** < 0.0001**	29 ± 13	19 ± 9	** < 0.0001**	29 ± 18	13 ± 9	** < 0.0001**
LCx	505 ± 119	490 ± 143	0.122	18 ± 7	26 ± 9	** < 0.0001**	28 ± 14	19 ± 8	** < 0.0001**	25 ± 13	13 ± 7	** < 0.0001**
AO	480 ± 113	481 ± 121	0.445	25 ± 8	30 ± 11	0.110	27 ± 12	18 ± 8	** < 0.0001**	28 ± 14	13 ± 7	** < 0.0001**

*HU* Hounsfield Unit, *CNR* contrast-to-noise ratio, *SNR* signal-to-noise ratio, *RCA* right coronary artery, *CTk* common trunk, *LAD* left anterior descending artery, *LCx* left circumflex artery, *AO* aorta, *MBIR* model-based iterative reconstruction, *IR* iterative reconstruction

*p*-values in bold represent statistical significant differences

Image noise was significantly lower in MBIR images compared with IR ones, in all the assessed vascular districts (all *p* < 0.05), except for the aorta (*p* = 0.110) (Table 3).

**Fig. 3:**
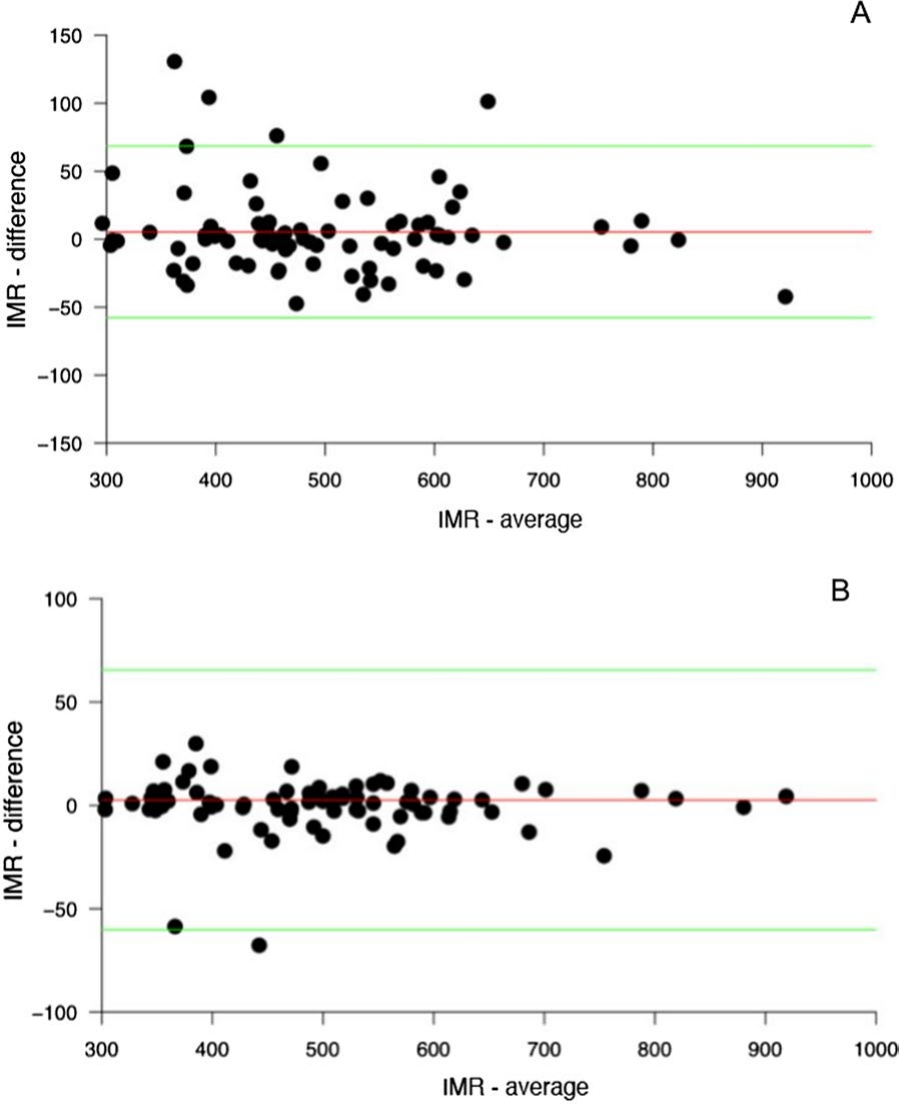
Comparison of Hounsfield unit (HU) with Bland–Altman analysis of right coronary artery (**a**) and left anterior descendant (**b**), between Reader 1 and Reader 2: red line shows the average of the differences, and green lines show the 95% limits of agreement (LoA)

The comparison of inter-reader evaluation of attenuation values and image noise didn’t show any bias, as reported in the Bland–Altman plot (Fig. 3).

#### CAD-RADS evaluation and inter-observer agreement

**Fig. 4:**
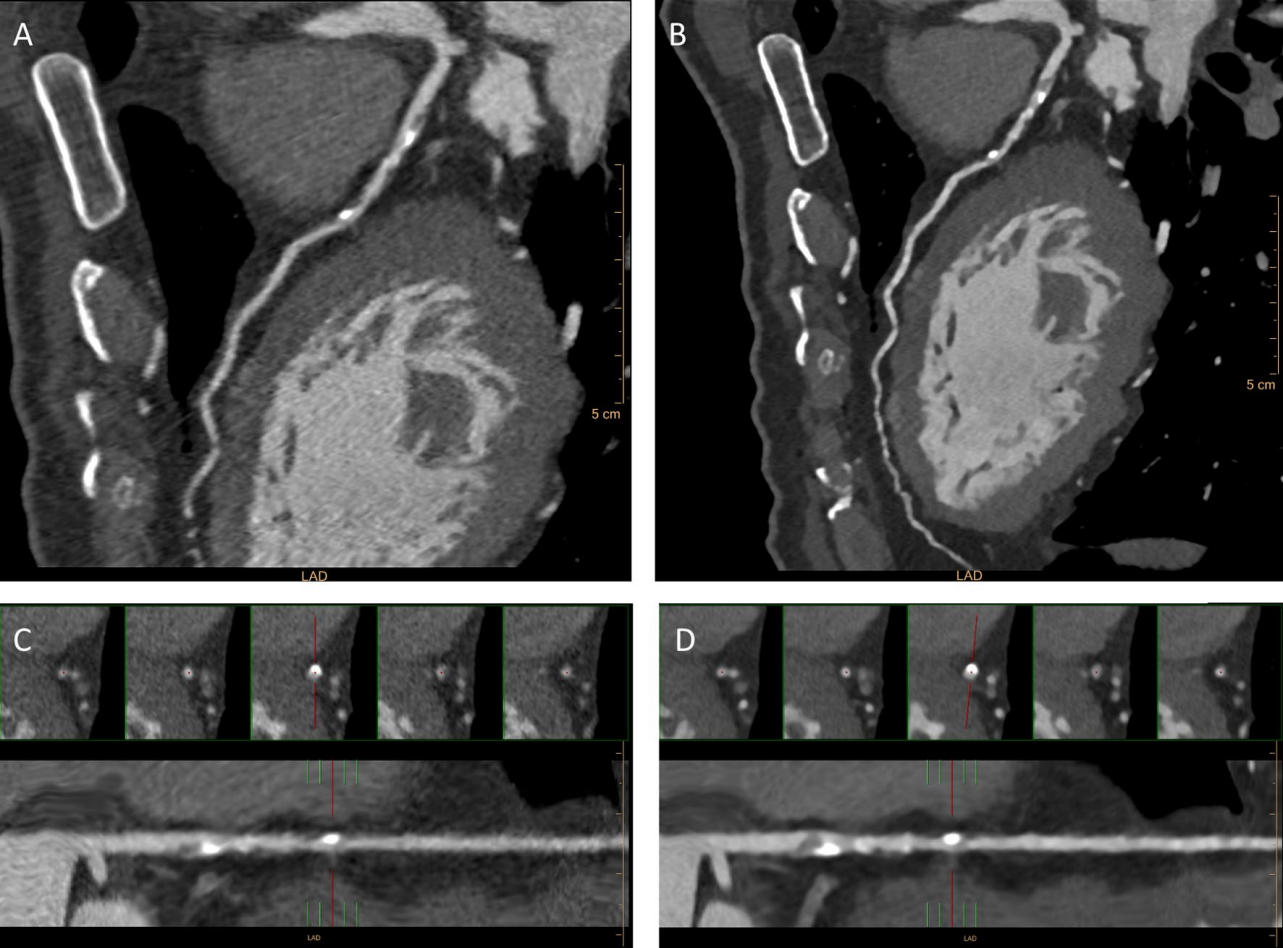
Evaluation of left anterior descending coronary artery of a single patient with both IR (**a** and **c)** and MBIR (**b** and **d)** reconstruction algorithm. The MBIR reconstruction better depicts the artery wall and the lesions in comparison with IR images because of lower image noise. **a–b** Curved reconstruction of the LAD artery showing multiple mixed atherosclerotic plaques in the proximal and intermediate tract. New model-based iterative reconstruction (MBIR) is also possible to evaluate the distal tract of the coronary artery analyzed. **c–d** Straight reconstructions of IR (**c**) and MBIR (**d**) images for a better evaluation of proximal atherosclerotic plaque, which determined luminal stenosis of 65–70% described as CAD-RADS 3

**Fig. 5:**
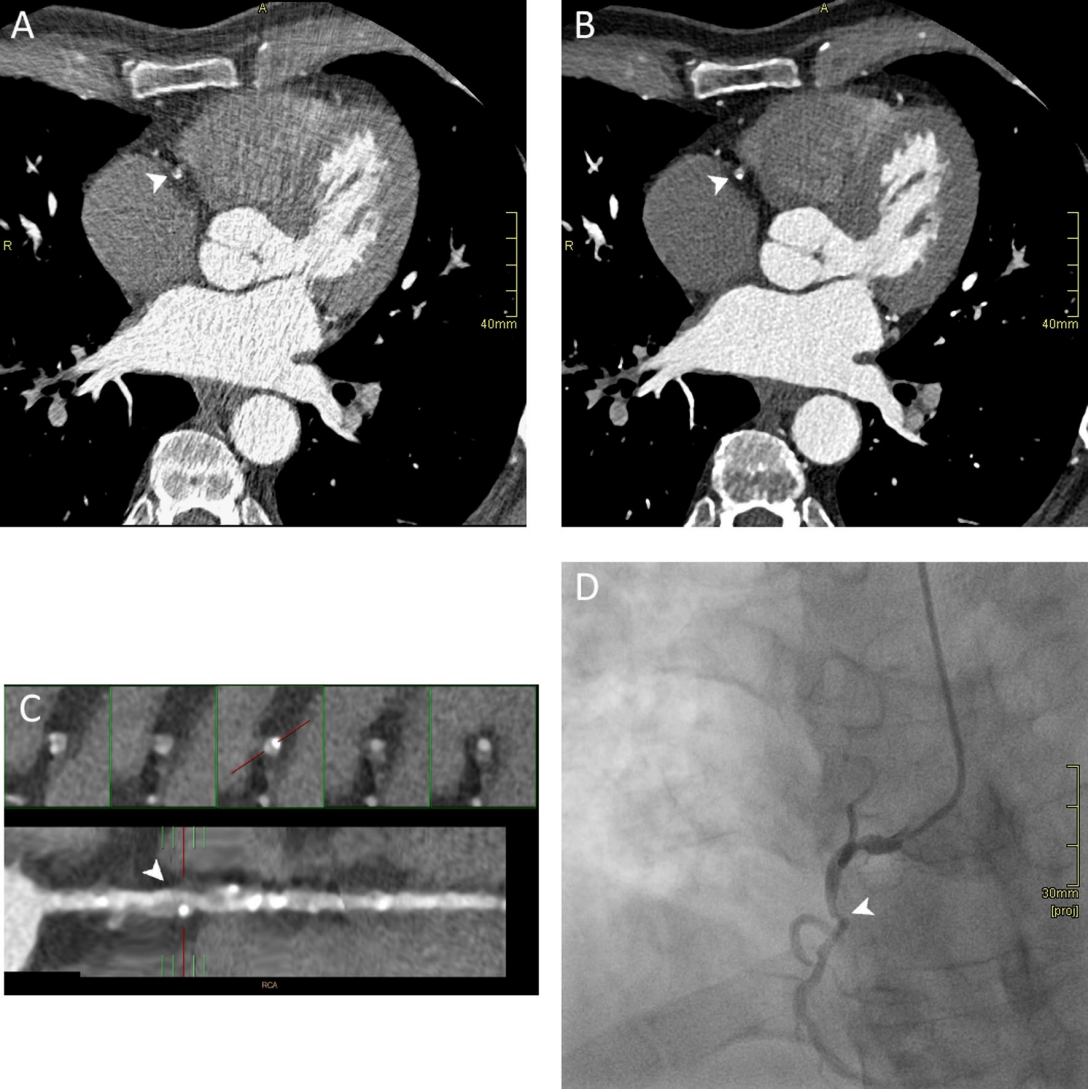
Comparison of CT images reconstructed with IR and MBIR algorithm and invasive coronary angiography (ICA) in a 65-year-old male patient with thoracic pain after exercise and a history of smoke and arterial hypertension. CT was acquired with 80-kV ECG-gated protocol (HR: 62 bpm). **a–b** Axial image reconstructions at the same level of the right coronary artery (RCA) show the presence of fibrous and calcified atherosclerotic plaque. MBIR image presented a lower noise level and higher contrast-to-noise ratio, leading to a better evaluation of the pathological findings. **c** Straight reconstruction which better shows the presence of multiple atherosclerotic plaques along the whole vessel. At the proximal tract, there is severe stenosis (arrowhead) quantified as 85% and reported as CADARDS 4A. **d** Invasive coronary angiography showing the stenosis reported in CT examination (arrowhead), confirmed as 80%

The reliability analysis between the two readers was almost perfect for all CAD-RADS standard categories, with a maximum value for CAD-RADS 0 (*k* = 0.965) and minimum value for CAD-RADS 3 (*k* = 0.801). When asking readers to evaluate CAD-RADS modifiers, we found a perfect agreement between the two readers for the 42 patients evaluated (*k* = 1.000). All data regarding the reliability analysis are reported in Table 4. Figures 4 and 5 report examples of CAD-RADS 3 and CAD-RADS 4A.

**Table 4:** CAD-RADS assessment categories between the two readers. The inter-observer agreement, computed with Cohen’s k statistics, was almost perfect for conventional standard categories and modifiers

CAD-RADS (*n*, %)	Reader 1	Reader 2	Agreement (*k*)
Standard categories (*N* = 160)			
0	39 (24.4)	40 (25.0)	0.965
1	29 (18.1)	27 (16.9)	0.912
2	24 (15.0)	20 (12.5)	0.885
3	13 (8.1)	19 (11.9)	0.801
4A	17 (10.6)	21 (13.1)	0.810
4B	12 (7.5)	10 (6.2)	0.910
5	26 (16.3)	23 (14.4)	0.840
Modifiers (*N* = 42)			
N	18 (42.8)	18 (42.8)	1.000
G	10 (23.8)	10 (23.8)	1.000
S	14 (33.4)	14 (33.4)	1.000

*N* = non-diagnostic study, *S* = presence of stent, *G* = presence of graft

### Radiation dose

A mean DLP of 63.9 ± 32.50 mGy*cm, mean CTDI of 4.32 ± 1.40 mGy, and a mean ED of 0.9 ± 0.47 mSv were reported.

## Discussion

This study aimed to confirm the feasibility and reproducibility of CAD-RADS and improve reporting consistency. Our results demonstrated an excellent inter-observer agreement for CAD-RADS assessment categories and modifiers, in line with the previous report from Maroules et al. [16]. Both early career and expert readers have proved a high concordance in CAD-RADS assessment, despite no experience in this scoring system. Szilveszter et al., using a reporting platform that determines automatically the CAD-RADS category [17], achieved slightly superior results compared to ours. The difference could be explained by different familiarity with the CAD-RADS lexicon. In our series, the major difference in terms of the inter-observer agreement was registered for CAD-RADS 0 and CAD-RADS 1, particularly in evaluating the small low attenuation plaque and, probably, due to the different expertise between the two readers.

In our series, we also evaluated the inter-observer agreement in the assessment of attenuation values within the coronary lumen obtaining satisfactory results without any significant bias.

When comparing model-based and iterative reconstruction algorithms, we obtained higher intra-luminal density values in each coronary vessel analyzed with MBIR compared to IR, which reached a statistically significant difference for CTk, RCA, and LAD. Moreover, the absolute attenuation values obtained in our study were similar to those reported by C.H. Park et al. [13], who used a similar iodine concentration contrast agent and 80 kV setting.

The same results were found regarding image noise: images reconstructed with MBIR reported a significantly lower noise in all vascular districts. Finally, SNR and CNR computed in MBIR images reached a statistically significant lower noise in comparison with IR ones, according to data reported in the literature [18].

In the last ten years, several prospective trials (firstly PROMISE and SCOT-HEART) and meta-analyses showed the high diagnostic value and the clinical utility of coronary computed tomography, leading to its endorsement as the first-line investigation in low-medium-risk patients [3, 11]. For these reasons, considering the high variability in CCTA reporting, a simple standardized scoring system for CAD classification was deemed necessary for subsequent clinical care and to provide management recommendations [19].

The CAD-RADS is a scoring system for CCTA that encompasses vessel stenosis grade, plaque morphology, and high-risk anatomy. It is based on the assessment of the severity of coronary arteries stenosis with a scale that ranges from CAD-RADS 0 for the total absence of coronary plaque or stenosis to CAD-RADS 5 for complete occlusion in at least one coronary vessel. Moreover, it investigates also sub-categories as modifiers, including high-risk plaque, presence of bypass graft or stent. The report applies to CCTA in patients with suspect or known CAD and contains recommendations for optimal patient management after cardiac CT, including further testing and therapeutic options [11, 12].

Several studies have proved that MBIR may simultaneously be able to reduce radiation dose and image noise with high-contrast and spatial-resolution improvement, resulting in a better assessment of the coronary artery lumen and improved visualization of plaques than the IR algorithm [20, 21]. According to the existing literature, our study remarks that the overall subjective image quality was better for MBIR images compared to IR ones. In particular, the application of new reconstruction algorithms can lead to a more accurate compilation of this scoring system, as well as in the evaluation of the small vascular structures, as for the coronary arteries.

This study has some limitations. Firstly, it included a small sample size, in particular regarding patients who underwent stenting or grafting procedures. Secondly, we did not evaluate the impact in terms of calcified plaque between the reconstruction of MBIR and IR. Indeed it is known that IR can reduce coronary calcification; in particular, it has been demonstrated that calcium scores were significantly lower for IR compared to FBP reconstructions [22]. Thirdly, the retrospective nature of the study can add unfitting selection bias. Finally, we can’t evaluate follow-up of patients (i.e., who underwent invasive angiography) due to the inability to collect these data.

In conclusion, the reliability analysis between readers underlines the diagnostic value of low-dose CCTA protocol, encouraging the use of the CAD-RADS lexicon in clinical practice. Moreover, the use of a model-based reconstruction algorithm allows a significant radiation dose reduction maintaining high diagnostic image quality and reducing the overall amount of image noise.

## Data Availability

All data generated or analyzed during this study are included in this published article.

## Abbreviations

ACR: American College of Radiology
ALARA: As low as reasonably achievable
CAD: Coronary artery disease
CAD-RADS: Coronary Artery Disease-Reporting and Data System
CCTA: Cardiac computed tomography angiography
CM: Contrast medium
CNR: Contrast-to-noise ratio
CT: Computed tomography
CTDI: Computed tomography dose index
CTk: Common trunk
DLP: Dose-length product
ED: Effective dose
FBP: Filtered back projection
ICA: Invasive coronary angiography
LAD: Left anterior descending artery
LCx: Left circumflex artery
MIP: Maximum intensity projection
MPI: Myocardial perfusion imaging
MPR: Multiplanar reconstructions
NASCI: North American Society of Cardiovascular Imaging
NICE: National Institute for Health and Care Excellence
RCA: Right coronary artery
SCCT: Society of Cardiovascular Computed Tomography
SNR: Signal-to-noise ratio
VR: Volume rendering
